# Lung and liver SBRT using helical tomotherapy — a dosimetric comparison of fixed jaw and dynamic jaw delivery

**DOI:** 10.1120/jacmp.v15i3.4664

**Published:** 2014-05-08

**Authors:** Leonie Rudofsky, Eleanor Aynsley, Sebastian Beck, Kai Schubert, Gregor Habl, Sonja Krause, Jürgen Debus, Florian Sterzing

**Affiliations:** ^1^ Department of Radiation Oncology University Hospital of Heidelberg Heidelberg Germany; ^2^ Department of Oncology and Radiotherapy The James Cook University Hospital Middlesbrough UK; ^3^ German Cancer Research Center Heidelberg Germany

**Keywords:** helical tomotherapy, stereotactic body radiation therapy, SBRT, dynamic jaws, treatment time

## Abstract

The purpose of the study was to evaluate the time effectiveness and dose distribution details of dynamic jaw delivery compared to the regular helical tomotherapy delivery mode in stereotactic body radiation therapy (SBRT) of liver and lung tumors. Ten patients with liver and ten patients with lung tumors were chosen to analyze the dose profiles and treatment times of regular helical tomotherapy delivery (2.5 cm field width) and new helical tomotherapy mode using dynamic jaw delivery with 5 cm field width. A median dose between 24 and 30 Gy was delivered in a single fraction. Regular helical tomotherapy took an average of 31.9 ± 6.7 min (lung SBRT) and 41.7 ± 15.0 min (liver SBRT). A reduction in delivery duration of 38.8% to 19.5 ± 2.9 min could be accomplished for lung irradiation (*p* < 0.05) and by 50.8% to 20.5 ± 6.0 min for liver SBRT (*p* < 0.05). Target coverage, as well as conformity and uniformity indices, showed no significant differences. No significant increase in organs‐at‐risk exposure could be detected either for lung or liver tumors. Therefore, use of new delivery mode with dynamic jaws improves treatment efficiency by reducing beam‐on time, while maintaining excellent plan quality.

PACS numbers: 87.55.D‐, 87.53.Ly, 87.55.N‐

## I. INTRODUCTION

The use of SBRT for radiation therapy of primary and metastatic tumors in different anatomical sites is becoming a standard of treatment. Radiosurgery was first described by Leksell^(1)^ and has its beginnings in irradiating intracranial lesions due to the favorable cranial anatomy. Immobilization is relatively easily to realize, internal motion of the organ is limited. External body radiosurgery is more challenging for radiation oncologists and technical support. Today, multiple studies show the feasibility of stereotactic single‐dose radiation therapy for extracranial targets, like singular inoperable liver metastases^2^, ^3^ and primary or secondary lung lesions^4^, ^5^, ^6^ with excellent local control. For IMRT/SBRT approach published literature has been limited, but demonstrates also favorable results.^7^, ^8^

The technical details of helical tomotherapy (HT) have been discussed in detail before.^9^, ^10^, ^11^ Nevertheless it wasn't primarily conceptualized for use of radiosurgery, and literature concerning SBRT with HT is poor. But results so far available could show excellent toxicity profiles in non‐small cell lung carcinoma (NSCLC) and lung metastases, even adjacent to critical structures.^(12,13,14^) While HT allows high dose conformity, it has often been criticized because of long treatment times,^(15)^ especially considering the evolution of other techniques of rotational therapy (VMAT) over the last years, offering very fast VMAT treatments for targets of low or intermediate complexity.^(16)^

New developments such as running‐start‐stop (RSS) in HT have the potential to speed up treatment time and to reduce dose penumbra of the superior and inferior border of the treatment volume. This technique is now commercially available as TomoEDGE (Accuray, Sunnyvale, CA). This mode uses (while couch speed is constant) a dynamic opening of the jaws during treatment delivery which reduces in this way the dose penumbra.^17^, ^18^, ^19^ In the past, the choice of a bigger field width always resulted in an increased penumbra as the dimension of the penumbra is defined by the chosen field width.^(10)^ Now, by using dynamic jaws and therefore reducing the dose penumbra, a bigger field width can be chosen, which results in shorter beam‐on times. The purpose of this planning study is to evaluate the time effectiveness and dose distribution details of dynamic jaw delivery compared to the conventional tomotherapy planning system in SBRT of liver and lung tumors.

## II. MATERIALS AND METHODS

### A. Patients

Ten patients with single liver metastasis and ten patients with lung metastasis or primary lung tumor were chosen for this planning study. All of these patients have been treated in our department by helical tomotherapy in regular helical tomotherapy delivery mode due to functional inoperability or refusal of surgery. Patients have been chosen sequentially after start of SBRT with helical tomotherapy in our department in 2006. An additional plan using dynamic jaws was created comparing planning details with the existent plans. The mean volume of the planning target volume (PTV) in liver patients was $185.3\pm 161.2\;{\text{cm}}^{3}$, widely varying from a minimum of $9.6\;{\text{cm}}^{3}$ to a maximum of $528\;{\text{cm}}^{3}$.

In lung patients, mean volume of the PTV was $21.8\pm 9.6\;{\text{cm}}^{3}$ ($6.7\;{\text{cm}}^{3}$ up to $36.5\;{\text{cm}}^{3}$).

### B. Radiotherapy planning

Helical tomotherapy planning was based upon a computed tomography (CT) scan with 3 mm slice thickness including contrast enhancement. Whole visible tumor was defined as GTV (gross tumor volume). Every patient received 4D CT for planning CT to evaluate breathing excursion and for defining the security margins for ITV definition (internal target volume). ITV was defined as the GTV expanded by the measured breathing excursions in the 4D‐scan. Depending on the localization, mobility of the tumor and therefore extent of breathing excursions varied. An additional safety margin of 4−6 mm, depending on the proximity to organs at risk (OAR), was added to create PTV. To minimize diaphragm motion and therefore breathing motion, an abdominal compression device was used, originally developed by Lax and Blomgren and colleagues at Karolinska Hospital in Stockholm.^(20)^ We did not use any further breath hold or real‐time tumor tracking methods. The patient was positioned in an individually shaped vacuum mattress that was rigidly linked to the stereotactic body frame. Median prescription dose was 24−30 Gy, calculated for 2 fractions, delivered in 1 fraction on a single day. This approach prevents potential problems by achieving a high dose in the target in a limited number of rotations. A second run of rotational therapy ensures the full application of the chosen dose. And in the absence of intrafractional motion, monitoring the break before delivering the second part of the fraction was used for an additional positioning control. This break has to be considered as intrafractional break due to the technical approach mentioned above.

### C. Dynamic helical tomotherapy delivery

The running‐start‐stop (RSS) mechanism, now called TomoEDGE, is providing flexible front (superior) and back (inferior) field borders. When the tumor comes into the radiation field projected by the front jaw, the back jaw stays at the superior border of the tumor until it reaches its maximum open position. Then the jaw stays in maximum open position and remains (along with the field width) constant while the tumor is moved through the fan beam until the inferior border of the tumor comes into the radiation field. Then the back jaw closes dynamically again.^(18)^

### D. Helical tomotherapy planning

Regular helical tomotherapy planning was done with the tomotherapy planning station version 3.1.2.9 (Accuray). Plans with field widths of 2.5 cm (regular delivery) were created. For the pitch, which is defined as the couch travel distance for a complete gantry rotation relative to the axial beam width at the axis of rotation, a value of 0.1 was chosen. This pitch was chosen to allow for a delivery of the fraction dose in one or two runs. A higher pitch resulted in the necessity for more repetitions. Optimization was started with an intensity modulation factor (IMF) (maximum leaf intensity divided by the average leaf intensity) of 2.0; toward the end of planning, it was decreased manually until 1.6−1.8 to accelerate gantry movement.

Dynamic jaw planning was performed with the research planning station version 6.1.0.10 (Accuray). For each patient, a dynamic jaw plan with maximum field width of 5 cm was created. Optimization with IMF was performed the same way as regular helical tomotherapy planning.

Dose constraints for both regular and dynamic jaw planning were the same. Maximum dose of esophagus was 14 Gy, heart 7 Gy, and spinal cord 8 Gy. The percent volume of lung tissue that receives 20 Gy should be <10%.

### E. Plan comparison and statistical analysis

Plan quality concerning the PTV was judged using $D_{99}$ and $D_{1}$ (dose to 99% and 1% of the target volume), ${\text{CI}}_{95}$ (conformity index; total volume covered by the 95% isodose divided by the volume of the PTV covered by the 95% isodose), UI (uniformity index; dose covering 5% of the PTV divided by the dose covering 95% of the PTV), and ${\text{TV}}_{95\%}$ (volume covered by 95% of the prescribed dose divided by the PTV volume). Prescription isodose volume (PIV) was defined as the volume covered by the 90% isodose. Furthermore Paddick Conformity $\text{Index}(={{\text{TV}}_{\text{PIV}}}^{2}/\text{TV}\times \text{PIV})$ was determined with the part of the target volume covered by the prescription isodose of 90% divided by the product of target volume and total volume covered by the prescription isodose of 90%.^(21)^ Sparing of organs at risk was judged by maximum and average dose, as well as the percentage of volume of liver or lung receiving 5, 10, or 20 Gy ($V_{5}$, $V_{10}$, $V_{20}$).

For statistical analysis, a two‐sided paired *t*‐test was used. A value of *p* < 0.05 was considered statistically significant.

## III. RESULTS

### A. Treatment time

**Figure 1:**
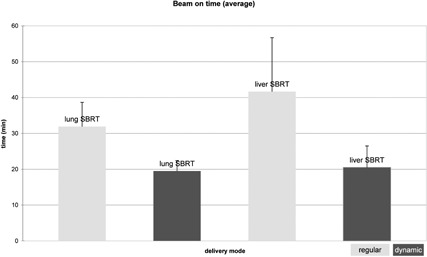
Average treatment times (min) for lung and liver SBRT for the regular helical tomotherapy delivery (with 2.5 cm field width) and dynamic jaw delivery (with 5.0 cm maximum field width) plans.

Regular HT took an average of 31.9±6.7 min (lung SBRT) and 41.7±15.0 min (liver SBRT). A reduction in delivery duration of 38.8% to 19.5±2.9 min could be accomplished for lung irradiation (p<0.05). Treatment time could be reduced by 50.8% to 20.5±6.0 min for liver SBRT (p<0.05, Fig. 1).

### B. Target coverage

Although for dynamic jaws planning a maximum field of 5 cm compared to 2.5 cm in regular helical tomotherapy delivery was used, dose distribution in the target volumes remained on a similar level. No significant differences could be detected for target coverage as indicated by ${\text{TV}}_{95\%}$ for regular and dynamic jaws and for uniformity indices, as well (Table 1).

**Table 1:** Plan characteristics for regular (with a 2.5 cm field width) and dynamic (with a 5.0 cm maximum field width) jaw delivery

*Target Volume*	*Parameter*	*Regular*	*Dynamic*	*p‐vah*
PTV lung	$D_{99\%}$ (Gy)	26.9±1.9	26.9±1.6	n.s.
	D_1%_(Gy)	30.1±1.8	30.1±1.9	n.s.
	UI	1.07±0.03	1.07±0.02	n.s.
	CI_95%_	1.20±0.18	1.35±0.28	n.s.
	TV_95%_	0.94±0.09	0.95±0.03	n.s.
	Paddick‐CI	0.68±0.14	0.59±0.13	n.s.
PTV liver	$D_{99\%}$ (Gy)	23.6±2.7	23.5±2.2	n.s.
	D_1%_(Gy)	28.5±1.9	27.9±2.3	n.s.
	UI	1.12±0.06	1.11±0.04	n.s.
	CI_95%_	1.10±0.09	1.10±0.09	n.s.
	TV_95%_	0.95±0.06	0.92±0.06	n.s.
	Paddick‐CI	0.79±0.09	0.76±0.08	n.s.

PTV=planning target volume; $D_{1/99\%}=\text{dose to}\;1\%/99\%\text{of the target volume}$; UI=uniformity index, dose covering 5% of the PTV divided by the volume of the PTV covered by the 95% isodose; ${\text{CI}}_{95\%}=\text{conformity index}$, total volume covered by the 95% isodose divided by the volume of the PTV covered by the 95% isodose; ${\text{TV}}_{95\%}=\text{volume covered}$ by 95% of the prescribed dose divided by the PTV volume; Paddick‐CI=Paddick Conformity Index; n.s.=not significant.

### C. OAR sparing

Analysis for organs at risk could not show any significant differences in regular and dynamic delivery mode. In lung SBRT the cardinal organ at risk, the contralateral lung, received both 0.6±0.3 Gy (regular) and 0.6±0.4 Gy (dynamic jaw) in average. $V_{5}$ of the ipsilateral lung was 18.6%±14.0% for regular and 18.5%±12.8% for dynamic delivery mode. In liver cases, $V_{20}$ of the liver was 11.3%±5.7% versus 12.7%±6.6% for regular and dynamic delivery, respectively (Table 2).

**Table 2:** Organs at risk and volume dose of liver and lung for regular (with a 2.5 cm field width) and dynamic (with a 5.0 cm maximum field width) jaw delivery

		*Lung SBRT*			*Liver SBRT*		
*OAR*	*Unit*	*Regular*	*Dynamic*	*p‐value*	*Regular*	*Dynamic*	*p‐value*
spinal cord±SD	Max (Gy)	3.3±0.8	3.4±1.0	n.s.	5.3±2.1	5.3±2.1	n.s.
heart±SD	Ave (Gy)	0.6±0.6	0.5±0.6	n.s.	4.1±2.4	5.0±3.5	n.s.
mediastinum±SD	Max (Gy)	6.3±2.4	7.5±2.1	n.s.	‐	‐	‐
esophagus±SD	Max (Gy)	4.1±0.7	4.6±1.0	n.s.	10.6±5.3	11.0±5.2	n.s.
liver							
$V_{5}\text{Gy}\pm \text{SD}$	(%)	‐	‐	‐	58.1±21.8	58.3±22.6	n.s.
$V_{10}\text{Gy}\pm \text{SD}$	(%)	‐	‐	‐	29.6±13.7	33.2±16.0	n.s.
$V_{20}\text{Gy}\pm \text{SD}$	(%)	‐	‐	‐	11.3±5.7	12.7±6.6	n.s.
lung							
$V_{5}\text{Gy}\pm \text{SD}$	(%)	18.6±14.0	18.5±12.8	n.s.	‐	‐	‐
$V_{10}\text{Gy}\pm \text{SD}$	(%)	7.8±6.3	8.6±6.8	n.s.	‐	‐	‐
$V_{20}\text{Gy}\pm \text{SD}$	(%)	2.5±1.7	3.0±2.0	n.s.	‐	‐	‐

OAR=organs at risk; SD=standard deviation; n.s.=not significant; $V_{5/10/20\;\text{Gy}}=\%$ of volume of liver or lung receiving 5/10/20 Gy.

Doses of spinal cord, heart, mediastinum, and esophagus for lung and liver SBRT did not differ significantly either (see Table 2).

## IV. DISCUSSION

In the present study, we investigated the potential of the new delivery mode of helical tomotherapy for SBRT for liver and lung cases. The treatment plan using dynamic jaws with a field width of 5 cm achieved the same quality of dose distribution compared to regular delivery mode with a field width of 2.5 cm by significantly shorter beam‐on times.

Helical tomotherapy has often been criticized for long treatment times; now beam‐on time for lung SBRT in this study using dynamic delivery mode resulted in 19.5±2.9 min, and in 20.5±6.0 min for liver SBRT. This means a time reduction compared to regular delivery between 39% and 51% for lung and liver SBRT, respectively. For patient setup, imaging, and repositioning another 7 min have to be added. This time remains unchanged, whether regular or dynamic mode is used. As far as this is comparable to other techniques of rotational therapy, nonetheless a reasonable time gain could be achieved by using the dynamic delivery mode in helical tomotherapy. In a study, treating primary or metastatic liver lesions with single dose of 25 Gy (total dose 75 Gy) overall treatment time including positioning, imaging, repositioning, and delivery using volumetric modulated arc therapy (VMAT) was about 16.2±1.7 min for the first and 12.4±1.5 min for the following fractions.^(22)^ VMAT used in early‐stage lung cancer treated with SBRT in 3 fractions of 18 Gy resulted in overall treatment time of about 20 to 25 min.^(23)^

With fixed field size, the regular dose delivery of helical tomotherapy produces typically a dose penumbra in the superior‐inferior direction due to the opening of the jaws by reaching the superior border of the target with its inferior border. Because of the continuous motion of the couch, the choice of the field width determines the dose gradient in longitudinal direction. This is, therefore, contributing to a dose elevation in tissue directly adjacent to the treatment volume.^24^, ^25^ The use of a 5 cm field width in regular delivery mode is, therefore, limited in clinical practice due to the significant dose exposure to healthy tissue above and below the PTV. By introducing dynamic jaw mode, a highly conformal longitudinal dose is produced compared to regular helical tomotherapy delivery, eliminating this problem.^(11)^ Now the primary beam is not irradiating the tissue before and after the tumor. The time for 1 fraction delivered in advanced mode remains equal to regular mode with corresponding field width, but the dose exposure to healthy tissue before and after the target is abandoned. So, the use of this largest field width of HT is going to be clinically feasible, meaning in practice a reduction of beamon time up to 51% by maintained plan quality, showed in this planning study. Accordingly, conformity index for lung cases varied only slightly from 1.2±0.18 (regular) to 1.35±0.28 (dynamic), which was not statistically significant and remained unchanged in liver cases with 1.1±0.09 for both techniques. Uniformity index was 1.07±0.03 in regular lung SBRT and 1.07±0.02 dynamic examples. Liver SBRT showed similar results with 1.12±0.06 (regular) and 1.11±0.04 (dynamic).

By using the dynamic approach the directly adjacent structures in superior and inferior direction can be spared more precisely, even with the use of the 5 cm field width, due to the advantages in longitudinal dose gradients. Nonetheless the dose distribution in lateral direction is less conformal due to the larger field width. The improvement of the longitudinal dose distribution is achieved at the cost of a larger lateral dose penumbra. Regarding the volume of liver and lung, respectively, receiving dose of 5, 10, and 20 Gy, we could not detect any significant differences as by different dose shaping in regular and advanced delivery overall organ dose exposure remains stable. The volume of the lung in this study receiving 5 or 20 Gy was <19% and <3%, respectively, for both delivery modes, being well below the clinically acceptable levels.

Whereas in conventionally fractionated radiotherapy the dose‐volume effect for developing a radiation‐induced pneumonitis is well established^(26)^ for SBRT, predictive parameters for radiation‐induced pneumonitis are rare so far. $V_{2.5}$ (relative volume of the ipsilateral lung exposed to doses of 2.5 Gy) has been identified to be an important DVH parameter with good correlation to development of pneumonitis The $V_{2.5}$ of 41.4%±8% was associated with development of pneumonitis, whereas values of 30.5%±13.8% were observed for patients without signs of pneumonitis.^(27)^

**Figure 2:**
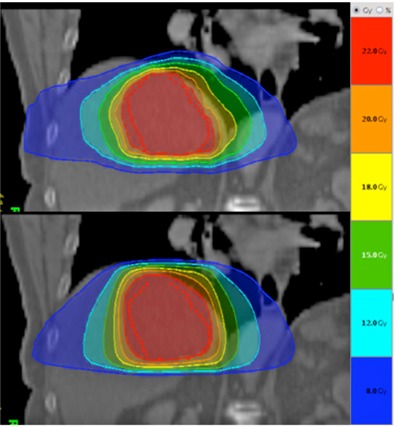
Comparison of dose distribution for patient with liver lesion receiving 24 Gy. Regular helical tomotherapy delivery (with 2.5 cm field width) above and dynamic jaw delivery (5 cm field width) below. Dynamic jaw dose distribution is more cylindrically shaped.

The use of this new technique may be limited in situations when the tumor volume is ajacent to an organ at risk in an oblique angle. Dynamic jaw delivery seems to be especially optimal for cylindrical target volumes where the dose distribution easily follows shape of PTV. In more elliptic shaped target volumes the dose distribution tends to exceed in horizontal direction when irradiating a more oblique angle (Fig. 2). Furthermore, by now producing an even sharper dose gradient in longitudinal direction one has to be aware that the risk of a geographical miss of the target is now back to the order of any other ungated or untracked radiotherapy technique. A dose penumbra in regular helical tomotherapy delivery yielded an extra safety net for longitudinal tumor motion.

## IV. CONCLUSIONS

Comparing new dynamic jaw with regular helical tomotherapy delivery mode in lung and liver SBRT showed reduction of beam‐on time by up to 51% without compromising target coverage or organs‐at‐risk sparing. By the improved longitudinal resolution, larger jaw width can be used and, therefore, significantly improve delivery efficiency.

The time‐sparing effect of this dynamic jaw delivery has been shown by our group for complex and large volumes in fractionated treatments previously. Now we also demonstrate that it enables an accelerated application of SBRT in both lung and liver lesions without compromising plan quality.

## ACKNOWLEDGMENTS

This work has been supported by the Faculty of Medicine, University of Heidelberg, Germany. The department of Radiation Oncology, University Hospital of Heidelberg has a research cooperation with Accuray.
